# TERT expression in human prostate tissue reveals a potential molecular switch in benign prostatic hyperplasia progression

**DOI:** 10.1007/s00345-025-06140-z

**Published:** 2025-12-11

**Authors:** Yajie Xu, Sheng Hu, Laurenz Berger, Philip Nicola, Philipp Weinhold, Alexander Tamalunas, Christian G. Stief, Martin Hennenberg, Patrick Keller

**Affiliations:** 1 Department of Urology, LMU University Hospital, LMU Munich, Munich, Germany; 2 Urologische Klinik und Poliklinik, Marchioninistr. 15, 81377 München, Germany

**Keywords:** Benign prostatic hyperplasia (BPH), Lower urinary tract symptoms (LUTS), Voiding symptoms, Telomerase reverse transcriptase (TERT), Genome-wide association study (GWAS), Genetic BPH

## Abstract

**Purpose:**

Benign prostatic hyperplasia (BPH) is a leading cause of voiding symptoms in aging men. Telomerase reverse transcriptase (TERT), a key regulator of cellular senescence and tissue remodeling, has not been systematically examined in human voiding symptoms, though genome-wide association studies suggested a role in genetic and sporadic BPH.

**Methods:**

Prostate tissues from patients undergoing laser enucleation for BPH were analyzed by RT-PCR. Clinical parameters were compared between TERT-positive and -negative patients. TERT expression in laser-enucleated tissues was tested for correlation with contractile smooth muscle receptors and the proliferation marker Ki67, and compared to tissues from radical prostatectomy for prostate cancer.

**Results:**

In a cohort of 100 patients undergoing laser enucleation of the prostate, we detected prostatic TERT mRNA in 81% of samples. Patients with TERT-positive tissues displayed clinically relevant higher symptom scores (22.5 vs. 17.4, *p* = 0.0407), and more pronounced post-void residual urine (80 vs. 142 ml). Prostate volume and free PSA showed no relationship with TERT expression. TERT expression correlated with expression of α_1A_-adrenoceptors (*p* < 0.0001), endothelin receptor A (*p* < 0.0001) and B (*p* < 0.0001), thromboxane receptor (*p* < 0.0001), thromboxane synthase (*p* = 0.0050) and Ki67 (*p* = 0.0181). TERT expression was 460-fold higher (*p* = 0.0015) in laser-enucleated than in periurethral prostatectomized tissues.

**Conclusions:**

TERT expression associates with higher symptom severity in laser-enucleated BPH patients, and correlates with targets imparting drug resistance. We propose that TERT acts as a molecular switch, marking a clinically more severe BPH phenotype. This finding raises the possibility of targeted intervention in BPH patients with TERT-driven pathology.

## Introduction

Genome-wide association studies (GWAS) and meta-analyses of candidate gene studies have repeatedly implicated telomerase reverse transcriptase (TERT) variants in benign prostatic hyperplasia (BPH) [1]. Yet, despite these genetic signals, no gene identified by BPH GWAS has undergone functional or clinical characterization in human tissue, and no real world data relating TERT with voiding symptoms in genetic or sporadic BPH are available [1]. This gap leaves the biological relevance of such genes unresolved and their therapeutic potential unexplored. BPH is an age-dependent disease, causing voiding symptoms by obstruction of the bladder outlet [2], in probably more than 600 million patients worldwide and with increases in global case numbers by 70.5% from 2000 to 2019, as a result of demographic transitions and with adherent demographic transitions [3, 4]. Management is primarily pharmacological in patients with mild to moderate symptoms, with or without early signs of progression, while surgery is advised for those with documented progression, complications or a high risk thereof, and for non-responders to drug therapy [5, 6]. α_1_-Adrenoceptor antagonists are the first-line pharmacological option, as smooth muscle contraction can drive obstruction independently, or alongside prostatic enlargement or tissue remodeling [7]. However, the success of drug treatment is limited to improvements of 50% and 30% of patients treated with α_1_-blockers are non-responders, possibly as non-adrenergic smooth muscle receptors account for drug resistance in symptom treatment [7]. The predictable developments of ageing societies call for identification of new drug targets, while the molecular drivers of BPH remain poorly defined and functional characterization of BPH-relevant prostate functions is still pending for almost all genes identified in GWAS [1].

TERT, a key regulator of cellular senescence and tissue remodeling, has not been systematically examined in the context of human BPH although it has been repeatedly implicated in independent genetic studies. Here, we report relationships of voiding symptoms, contractile smooth muscle receptors and the need for surgery in patients undergoing holmium or thulium laser enucleation (HoLEP, ThuLEP) for BPH with TERT expression detected by real-time polymerase chain reaction (RT-PCR).

## Methods

### Human tissues, patients

Prostate tissues were collected from HoLEP and ThuLEP for BPH (100 patients), and from radical prostatectomy (rPx) for prostate cancer (20 patients). This study was carried out in accordance with the Declaration of Helsinki of the World Medical Association and has been approved by the ethics committee of Ludwig Maximilian University, Munich, Germany (approval number 22–0608). Written informed consent was obtained from all patients. All surgeries were performed at the Department of Urology of the LMU University Hospital.

The laser-enucleated cohort included patients catheterized for urinary retention prior to surgery (*n* = 38) and patients without catheterization (*n* = 61), and a patient without information on catheter status in the clinical records. From a total of 100 participating patients undergoing laser enucleation, international prostate symptom scores (IPSS) were available from 61 patients (57 without catheterization, 4 catheterized), data for post-void urine volume (PVR) from 53 patients (all without catheterization), for free prostate-specific antigen (PSA) from 99 patients, and prostate volume (PV) from all 100 patients. Data from tissue analyses by RT-PCR and clinical data were pseudonymized, and merged data finally anonymized. In contrast, tissues from rPx were anonymized during sampling, and no clinical data were collected or analyzed for this study.

At our department, the median age of patients undergoing prostatectomy for prostate cancer is 66 years (*n* = 4,003 and *n* = 5,800 in two large cohorts) [8, 9]. Among 5,489 such patients, 49.7% reported an IPSS ≥ 8, defined as “LUTS [lower urinary tract symptoms] in need of treatment”, with a mean score of 14, median age of 67 years) and a median prostate volume of 56 ml [9]. The remaining 50.3% had an IPSS < 8 (3 on average, 65 years) and a prostate volume of 49 ml [10]. In another dataset of 5,899 patients prostatectomized at our department, 99 had received preoperative 5α-reductase inhibitors, reported a median IPSS of 7 and had a median prostate volume of 61.5 ml [9]. The 5,800 without such medication had a median IPSS of 11 and a median prostate volume of 52 ml [9]. In this age group (60–69 years), histological BPH is present in approximately 60–70% of men [2], and “clinical BPH” (IPSS > 7, Q_max_ <15 ml/s) in up to 35% [2], whereas concomitant histological BPH is found in approximately 80% of prostate cancer patients [11, 12].

In contrast, men treated by laser enucleation typically present with more advanced BPH. In 1,593 patients undergoing HoLEP for LUTS due to benign prostatic obstruction (BPO), the median IPSS was 20, the Q_max_ 10 ml/s, the PVR 90 ml, and the age 71 years at our department (2018–2021) [13]. In 606 propensity-matched HoLEP patients at our department (2017–2020, three-lobe and one-lobe enucleation, each *n* = 303), the median IPSS was 21 in each group, the Q_max_ 10 and 9 ml/s, the PVR 80 and 100 ml, and the age 70 and 71 years [14]. In 852 age-stratified HoLEP patients, the median IPSS ranged from 17 to 19, the PVR from 60 to 100 ml, and the median Q_max_ mounted consistently to 11 ml/s in the three groups (PV ≤ 60 ml, > 60 and < 120 ml, ≥ 120 ml) at our department (2014–2018) [15]. According to established IPSS staging criteria, scoring of 0–7 indicate mild voiding symptoms, 8–19 moderate, and 20–35 severe symptoms [16]. Thus, men undergoing rPx for prostate cancer generally fall into the mild-to-moderate range, whereas those treated with laser enucleation usually present with moderate-to-severe symptoms, often accompanied by complications, consistent with advanced BPH.

### Human prostate tissues from radical prostatectomy

Periurethral prostate tissues were dissected by a pathologist within 30–60 min after surgical removal of the prostate, after opening by a longitudinal cut from the capsule to the urethra, and visual inspection of the intersections for tumor infiltration. If tumor infiltration did not exclude sampling, tissues were cutted from the periurethral zone, and samples were shock frozen in liquid nitrogen within one hour. Macroscopically visible tumor infiltration was rare in this region (affecting less than 1% of prostates), consistent with the typical predominance of tumors in the peripheral zone [17, 18]. For transport and interim storage, tissues and organs were kept in Custodiol^®^ solution.

### Human prostate tissues from laser enucleation

HoLEP and ThuLEP were carried out using a three-lobe enucleation approach, as recently detailed [19]. After retrieval of the morcellated tissue fragments from the bladder, samples were promptly placed in Custodiol^®^ solution for transport and interim storage, and during selection of shreds for use in further analyses. Samples were shock frozen within one hour after tissue extraction.

### RT-PCR

RNA was isolated from frozen tissues using the RNeasy Mini Kit (Qiagen, Hilden, Germany) based on the manufacturer’s instructions. For isolation, 30 mg of frozen tissues was homogenized using the FastPrep^®^-24 system with matrix A (MP Biomedicals, Illkirch, France). RNA concentrations were measured spectrophotometrically. cDNA was synthesized using 500 ng of isolated RNA using a Reverse Transcription System (Promega, Madison, WI, United States). RT-PCR was performed with a Roche Light Cycler (Roche, Basel, Switzerland). Primers (with RefSeq accession number for detected transcripts) for human telomerase reverse transcriptase (TERT, NM_198253), α_1A_-adrenoceptor (ADRA1, NM_033303), endothelin receptor A (ENDRA, NM_001957), endothelin receptor B (ENDRB, NM_003991), thromboxane A2 receptor (TBXA2R, NM_001060), thromboxane synthase (TBXAS1, NM_001061), Ki67 (MKI67, NM_002417) and glyceraldehyde-3-phosphate dehydrogenase for housekeeping (GAPDH, NM_002046) were purchased from Qiagen (Hilden, Germany). The PCR reaction volume was 10 µl, which included 5 µl FastStart DNA MasterPlus SYBR Green I (Roche, Basel, Switzerland), 1 µl primer, 1.5 µl PCR grade water and 2.5 µl sample. Denaturation was performed at 95 °C for 10 min, and amplification with 40 cycles, each including 10 s at 95 °C, 10 s at 60 °C, 15 s at 72 °C. PCR product quality was demonstrated by post-PCR melt curve analysis. All samples were determined in duplicate and presented as means of these two replicates. Results were expressed using the 2^−ΔCt^ method, where number of cycles (Ct) at which the fluorescence signal exceeded a defined threshold for GAPDH was subtracted from Ct values for targets (Ct_target_-Ct_GAPDH_ = ΔCt), and values were calculated as 2^−ΔCt^ and normalized to the mean values of corresponding controls.

### Data analyses

Data analyses, including Pearson correlation analyses were performed using GraphPad Prism 6 (GraphPad Software Inc., San Diego, CA, USA). Groups were compared by unpaired Student’s t-tests if data were normally distributed in each group, or by Mann-Whitney test if data showed no normal distribution in a least one of both groups. P values < 0.05 were considered statistically significant. Tests for normality were performed by D’Agostino & Pearson omnibus normality tests. Data in the text are reported as means or mean differences with 95% confidence intervals (95%CI). The wide range of 2^−ΔCt^ values (across several orders of magnitude) required conversion to logarithmic values to allow meaningful visualization in linear regression diagrams, so that TERT-negative values (2^−ΔCt^=0) were excluded in linear regression diagrams. However, calculation of Pearson r and p values included the whole populations. The present study and analyses have exploratory character and were not designed to test a pre-specified or statistical null hypothesis. Features of a typical hypothesis-testing study are lacking, including the definition of a tested hypothesis, or a study plan based on the biometric calculation of group sizes. Owing to the exploratory study design, p values reported here are descriptive, but not hypothesis-testing.

## Results

### TERT expression and BPH-specific clinical parameters

**Fig. 1:**
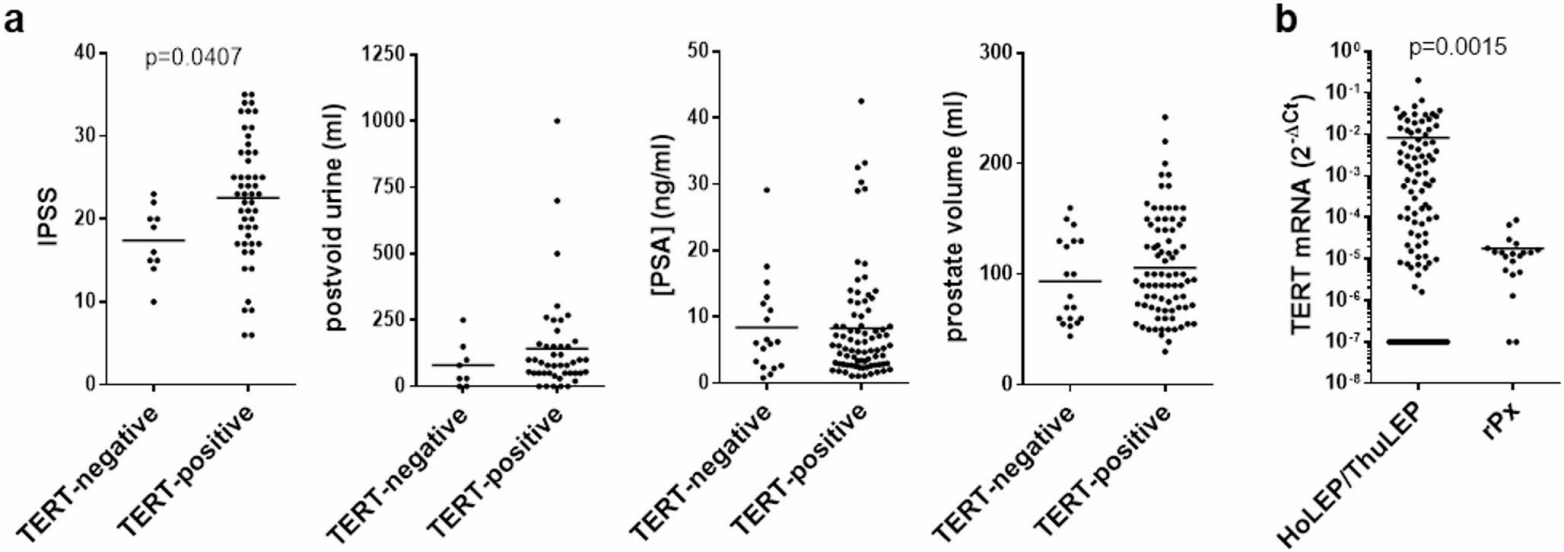
Voiding symptoms, post-void residual urine, free PSA and prostate volume in relation to prostatic TERT expression in patients undergoing laser-enucleation for BPH. **(a)** Tissues from HoLEP and ThuLEP (100 patients) were analyzed for TERT expression by RT-PCR, and international prostate symptom scores (IPSS) (*n* = 61 with available IPSS values), post-void urine volume (*n* = 53 with available values), free PSA (*n* = 100) and prostate volume (*n* = 100) were grouped for undetectable (TERT-negative) and detectable (TERT-positive) TERT expression. **(b)** TERT expression in prostate tissues from patients undergoing laser-enuclation for BPH, and in periurethral protsate tissues from patients undergoing radical prostatectomy for prostate cancer without prior surgery for BPH (*n* = 20). Groups were compared by Mann-Whitney tests, except IPSS values in (a), which were compared by unpaired Student’s t-test

TERT transcripts were detectable in laser-enucleated prostate tissues from 81 (TERT-positive), and undetectable (TERT-negative) from 19 of 100 examined patients. IPSS were higher with TERT-positive compared to TERT-negative prostate tissues (22.5 [95% CI 20.4–24.6] vs. 17.4 [14.5–20.3], mean difference 5.1 [0.2–10], *p* = 0.0407, *n* = 61 patients with available IPSS values) (Fig. 1a). TERT-positive patients tended to a higher PVR than TERT-negative patients (80 [8-152] vs. 142 [87–192] ml, mean difference 62 [-72 to 197] ml, *p* = 0.2646). PV (94 [75–113] ml TERT-negative, 106 [96–116] ml TERT-positive, *p* = 0.3621) and free PSA (8.3 [4.8–12] ng/ml TERT-negative, 8.3 [6.4–10] ng/ml TERT-positive, *p* = 0.8484) showed no relationship with TERT expression (Fig. 1a). In comparison, tissues from laser enucleation for BPH showed 460-fold higher TERT expression (*p* = 0.0015) than periurethral, non-cancerous prostate tissues from patients undergoing radical prostatectomy for prostate cancer without prior surgery for BPH, i.e. without signs of BPH progression and without BPH-related complications (Fig. 1b).

### Correlation of TERT expression with contractile receptors and Ki67

**Fig. 2:**
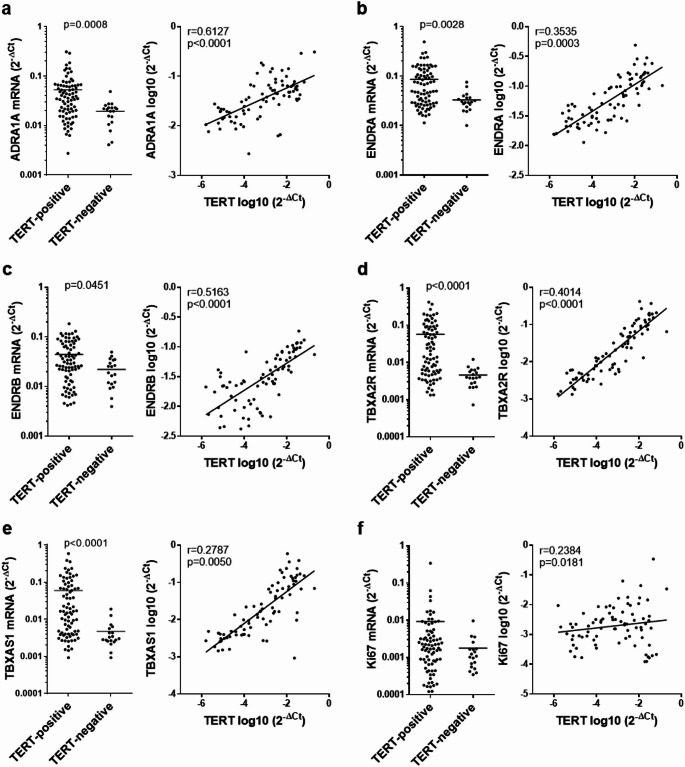
Expression of smooth muscle contraction-related receptors and Ki67, and correlation with TERT levels in laser-enucleated prostate tissues. Tissues from HoLEP and ThuLEP (100 patients) were analyzed by RT-PCR for TERT, and α_1A_-adrenoceptors (ADRA1) **(a)**, endothelin receptor A (ENDRA) **(b)**, endothelin receptor B (ENDRB) **(c)**, thromboxane A_2_ receptor (TBXA2R) **(d)**, thromboxane synthase (TBXAS1) **(e)** and Ki67 **(f)**. The r and p values refer to the whole population (*n* = 100, including 81 TERT-positive and 19 TERT-negative samples), which are indicated in diagrams and were calculated by Pearson correlation of 2^−ΔCt^ values. Diagrams for linear regression include only values from TERT-positive samples, as meaningful visualization required logarithmic conversion, which was not possible for TERT-negative samples (2^−ΔCt^=0). Groups were compared by Mann-Whitney tests, and analyzed by Pearson correlation analyses

TERT-positive tissues from laser enucleation showed markedly higher mRNA expression of smooth muscle contraction-related receptors, including α_1A_-adrenoceptors (2.7-fold of TERT-negative, *p* = 0.0008), endothelin receptor A (2.6-fold, *p* = 0.0028), endothelin receptor B (2-fold, *p* = 0.0415) and thromboxane A_2_ receptor (11.4-fold, *p* < 0.0001) (Fig. 2a-d). Thromboxane synthase (12.7-fold, *p* < 0.0001), and the proliferation marker Ki67 (5.2-fold) were elevated as well (Fig. 2e, f). TERT levels (including TERT-negative samples, with 2^−ΔCt^=0) correlated with α_1A_-adrenoceptor mRNA (*r* = 0.6127, *p* < 0.0001), endothelin receptors A (*r* = 0.3535, *p* = 0.003) and B (*r* = 0.5163, *p* < 0.0001), thromboxane A_2_ receptor (*r* = 0.4014, *p* < 0.0001) and thromboxane synthase (*r* = 0.2787, *p* = 0.0050), and partly with Ki67 (*r* = 0.2384, *p* = 0.0181) (Fig. 2).

## Discussion

Our findings suggest that TERT expression associates with higher symptom severity in laser-enucleated BPH patients and correlates with genes suspected to impart drug resistance, and with a proliferation marker. The difference in IPSS between patients with undetectable and detectable prostatic TERT levels is likely to be clinically relevant, as the threshold for patient perception is around 3 points [20]. Symptoms in laser-enucleated BPH patients are mostly refractory to treatment with α_1_-blockers or tadalafil [5, 21, 22]. Medication-refractory voiding symptoms in BPH have been provisionally explained by non-adrenergic mediators, including endothelin and thromboxane inducing a maximum prostate smooth muscle tone by activation of their cognate receptors, which is resistant to α_1_-blockers [7]. However, this explanation must be considered preliminary and is still under discussion, as definitive evidence is currently lacking. The higher TERT expression in laser-enucleated tissues compared to prostatectomized tissues without prior BPH surgery suggests an involvement of TERT in BPH progression to the need for surgery. We propose that TERT acts as a molecular switch, marking a clinically more severe BPH phenotype. This finding confirms a previously suspected but unproven role of TERT in sporadic and genetic BPH, and raises the possibility of targeted intervention in BPH patients with TERT-driven pathology.

TERT polymorphisms have been repeatedly related with symptomatic, genetic BPH, while our data are to the best of our knowledge the first linking TERT expression with voiding symptoms and with the need for surgery for BPH. A meta-analysis of 74 candidate gene studies identified a significant, though weak association of the TERT polymorphism rs2736098 with lower urinary tract symptoms (LUTS) in BPH (OR 1.25 [1.04–1.2]) [23]. Later, a GWAS identified the TERT variant rs2853677, associating significantly, but again weakly (OR 1.13 [1.1–1.16]) with medical or surgical treatment of LUTS in BPH [24]. However, no cumulative allele scores for multiple TERT variants or any polygenic risk scores have been calculated for BPH, and no functional work has connected TERT expression to disease features [1]. GWAS on BPH are still awaiting their first systematic review, standing in contrast to prostate cancer, where first meta-analyses of GWAS became available more than 15 years ago. TERT is the core component of telomerase, and can maintain telomere length and suppress apoptosis, thereby supporting cell survival and proliferative capacity [25]. In the prostate, elevated telomerase activity and TERT expression were found in stem-like and progenitor cell populations, but not yet in somatic cells [26]. Luminal progenitor cells may be a key contributor to epithelial hyperplasia in BPH [27, 28]. The glandular epithelium in BPH is composed of luminal and basal cells [29]. Both basal and luminal cells arise from telomerase-active luminal progenitor cells, suggesting that targeting telomerase activity, rather than hormonal pathways alone, may offer a novel therapeutic approach in BPH. Our findings extend suspected TERT functions in BPH to stromal cells, opening a potential link to smooth muscle contraction and drug-refractory symptoms. Consequently, an alternative explanation for the correlation of TERT expression with the other genes noted in our experiments may be co-expression as a feature of a dominant cell population. This and other possibilities must be considered as well, though the possibility that TERT triggers expression of other critical genes is also plausible.

Our results highlight a possible therapeutic potential of targeting TERT, but do not provide mechanistic insights into disease pathophysiology. Further analyses such as cell type-specific localization of TERT (stromal vs. epithelial compartments), or pathway and functional data may be subject of follow-up studies. In addition, a number of studies have shown non-equivalence of TERT expression and function [30]. Specifically and exemplarily, a number of splice variants are known to be inactive, resulting in non-functional TERT [30, 31], while our qPCR primer sequence detects a conserved region located upstream of the α (exon 6) and β (exons 7–8) splice sites. Our assay therefore does not specifically detect the functional full-length (+α + β) isoform. Accordingly, the measured TERT mRNA levels should be interpreted as an indicator of transcriptional activation of the TERT locus rather than as a surrogate for telomerase activity. In fact, substantial fractions of samples expressing TERT may lack telomerase activity [32]. In renal tissues, TERT mRNA expression was not sufficient to produce active telomerase enzyme, directly reflecting possible non-equivalence of expression and enzymatic function [33]. Finally, TERT may exert canonical functions, by telomer regulation, but may affect cell functions also by non-canonical, telomer-independent functions, by different intracellular signaling pathways [34, 35]. Future studies involving splice variant-specific assays, direct telomerase activity measurements and pathway analyses are warranted to further clarify the functional relevance of these transcriptional changes.

Our data links TERT expression with greater LUTS severity and a surgical BPH phenotype, independent of known genetic variants. However, a causality is not established by our data. TERT may serve as a marker of more severe BPH phenotypes, but whether TERT upregulation is a driver of disease progression or a secondary consequence of advanced pathology remains unresolved. Nevertheless, the observed differences in IPSS are of clinically relevant size and paralleled by trends in PVR. The dramatic upregulation of TERT in surgically treated versus mild, uncomplicated BPH suggests that TERT may contribute to progression toward surgery-requiring disease. However, periurethral tissues from prostate cancer patients reflect a population with specific biological and pathological characteristics, limiting their use as controls in our study. Ideally, non-cancer, non-surgical controls would provide a more appropriate comparator, but such tissues are hardly, if at all accessible. The molecular associations with adrenergic and non-adrenergic smooth muscle receptors, and with a proliferation marker, align with potential determinants of bladder outlet obstruction and drug resistance. Contraction, proliferation and growth in the prostate are considered as determinants of bladder outlet obstruction in BPH, and are the targets for medical treatment [2, 5]. Together, our findings also underscore the gap between genetic associations and functional validation for BPH-related genes [1]. Given the predictable rise in BPH burden with aging populations, TERT and other GWAS-identified genes warrant targeted functional studies to assess their roles in pathophysiology and potential as therapeutic targets, beyond specific variants.

## Data Availability

All data supporting the findings of this study are available within the paper.
